# Supplementary material for: Decreasing trend of imported malaria cases but increasing influx of mixed P. falciparum and P. vivax infections in malaria-free Kuwait
Source: PLoS One. 2020 Dec 11;15(12):e0243617. doi: 10.1371/journal.pone.0243617 (PMC7732060; doi:10.1371/journal.pone.0243617)
Supplement: S1 File — (DOCX) [file pone.0243617.s001.docx]

**Supplementary data: Figure 1.**


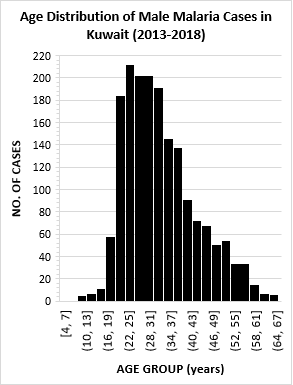

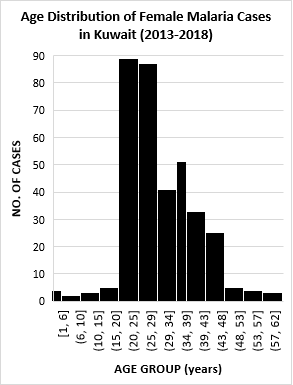


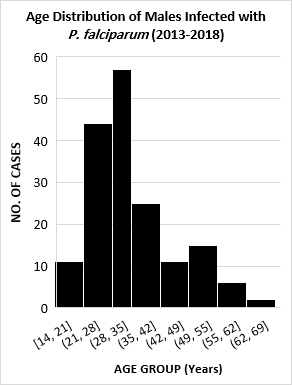

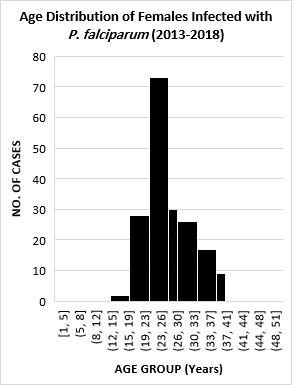


**Figure 1.** Histograms showing age distribution of all malaria cases by gender (males, top left; females, top right) and P. falciparum malaria cases (males, bottom left; females, bottom right) in Kuwait (2013-2018) (P<0.001).

**Supplementary data:**

**Figure 2**

**Figure** **2.** A line graph showing the monthly distribution of reported P. falciparum + P. vivax mixed infection, P. falciparum, and P. vivax malaria cases in Kuwait (2013-2018).
