# Supplementary material for: Decreasing trend of imported malaria cases but increasing influx of mixed P. falciparum and P. vivax infections in malaria-free Kuwait
Source: PLoS One. 2020 Dec 11;15(12):e0243617. doi: 10.1371/journal.pone.0243617 (PMC7732060; doi:10.1371/journal.pone.0243617)
Supplement: Attachment — Submitted filename: Reviewers comment on PONE-D-20-30612.docx [file pone.0243617.s002.docx]

26 October 2020

To: Editorial Office, PLOS ONE

Dear Editor,

Thank you very much for giving me the opportunity to review the manuscript “PONE-D-20-30612” titled as “Decreasing trend of imported malaria cases but increasing influx of mixed P. falciparum and P. vivax infections in malaria-free Kuwait”. The authors sought to characterize the incidence of imported malaria infection and evaluated the impact of malaria preventive measures in Kuwait using a retrospective chart review over a 6-year period. The authors note a total of 1913 (25.9%) malaria cases, 81.5% of which were among male subjects. Also, majority of malaria cases (1895; 99.1%) were detected among expatriates from 38 malaria-endemic countries. Overall, the paper is nicely written. However, there are spaces for improvement. I have few comments:

Methods:

- What prevention and control measures were in place? Was there overall malaria report from other source e.g. Govt. Health Department to understand the limitations of the findings?
- Data collection paragraph does not detail the procedure for data abstraction. There is need to describe how the facility collected malaria information. Who collected the samples? All these relevant information should be clearly indicated in the “M & M”.
- How was consistency assessed? It is important to include data with a few missing variables to avoid bias, please clarify how many records were excluded due to incomplete data.
- How malaria positive patients were administered?

Discussion:

- Reference for lines 252-253
- Please re-read for grammar edits pp. 12, line 255 insert comma (,) after “On the contrary,…”

Thank you very much.

*With regards,*

*Araya Gebresilassie (Ph.D)
Department of Zoological Sciences, Addis Ababa University*
