# Supplementary material for: Decreasing trend of imported malaria cases but increasing influx of mixed P. falciparum and P. vivax infections in malaria-free Kuwait
Source: PLoS One. 2020 Dec 11;15(12):e0243617. doi: 10.1371/journal.pone.0243617 (PMC7732060; doi:10.1371/journal.pone.0243617)
Supplement: Attachment — Submitted filename: Responses to Reviewers.docx [file pone.0243617.s003.docx]

Dated: 5^th^ November, 2020

Dear Ms Carvalho

Academic Editor, PLOS ONE

**Re: PONE-D-20-30612**

*‘Decreasing trend of imported malaria in cases but increasing influx of mixed P. falciparum and P. vivax infections in malaria-free Kuwait’.*

With reference to above manuscript, I take this opportunity to thank you and both the reviewers, Prof. Llanos-Cuentas and Prof. Gebresilassie on their thorough review and excellent suggestions to further improve the quality of the manuscript.

We have made the appropriate changes and edits as suggested by the Reviewers. The itemized responses to their queries are follows:

Both the reviewers have agreed and have no suggestion/comments to the following questions:

1. Is the manuscript technically sound, and do the data support the conclusions?
2. Has the statistical analysis been performed appropriately and rigorously?
3. Have the authors made all data underlying the findings in their manuscript fully available?
4. Is the manuscript presented in an intelligible fashion and written in standard English?

**Review Comments to the Author**

**Reviewer 1:**

**Query 1 & 2:** *Specify the malaria treatment scheme used in Kuwait & comment on 28-day treatment with primaquine*

**Answer:** We have edited the text as follows on Page 13, lines 259-270 and included two additional references (#35, 36).

All mixed infections with *P. falciparum + P. vivax* were treated with artemether+lumefantrine, given twice a day for 3 days. This treatment was followed by treatment with the standard dose of primaquine (0.25 mg/kg/day) for 28 days to treat hypnozoite stage of the *P. vivax.* Though the WHO has also recommended a higher dosage of primaquine (0.5 mg/kg/day) for 14 days, however, we used the standard dose for primaquine to minimize the side effect of hemolysis especially in patients with glucose‐6‐phosphate dehydrogenase (G6PD) deficiency. However, up to 20% of the mixed *Plasmodium* cases showed relapse of mixed *Plasmodium* infection 3-4 months after therapeutic/radical anti-malarial treatment with no travel history to the Indian sub-continent. A number of studies have reported in mixed malaria, an increase in relapse rates by *P. vivax* following treatment of *P. falciparum* [35]. Though, the most common cause of *P. vivax* relapse is poor adherence to treatment or inappropriate dose, however, failure in treatment due to impairment in CYP2D6 enzyme is also being investigated [36].

**Query #2:** *make some comment on the possibility of future use of tafenoquine*

**Answer:** Though the use of Tafenoquine as a single-dose (300 mg) therapy for *P. vivax* relapse prevention was discussed but not yet approved by the Ministry of Health, Kuwait, considering its safety challenge in causing severe hemolytic anemia in people with G6PD deficiency. However, we hope that the Ministry of Health may approve its use soon considering rise of *P. vivax* relapses following treatment of mixed malaria infections.

**Reviewer 2:**

**Query 1 & 2:** *What prevention and controlmeasures were in place & What was the detail on Data collection?*

**Answer:** We have edited the text as follows on Page 4, lines 74-86 under Materials & Methods.

Malaria infection is a notifiable disease in Kuwait. Though all migrant workers from malaria endemic countries entering Kuwait for the 1^st^ time are required to carry a recent malaria-free certification issued by the designated Diagnostic Laboratories in their home countries, however, they are also screened for malaria infection at the Ports and Borders Health Centers in Kuwait. Kuwait is a small country consisting of six administrative governorates. All malaria positive cases are then referred to the Malaria Reference Laboratory, Infectious Diseases Hospital (MRL, IDH), Kuwait for treatment and follow up. In addition, any resident in Kuwait suspected of malaria infection is referred to the MLR, IDH for confirmation and therapeutic management. For all cases, information on civil identification number, age, gender, nationality, referring hospital, date of sample collection, major clinical symptoms, recent travel history and test results are recorded in the MLR, IDH registry logbooks. The epidemiologic and demographic data of all cases was collected from the registry logbooks of MRL, IDH during the period January 2013 to December 2018.

**Query 3:** *How was consistency assessed?*

**Answer:** As mentioned above, the epidemiological data for all referred cases was recorded in the MLR, IDH registry logbooks. There were just 4-5 cases with missing information on their nationality and/or travel history, which we did not include in our data as the number was very small.

**Query 4:** *How malaria positive patients were administered?*
**Answer:** Please refer to our answer to query # 1 & 2 for the 1^st^ reviewer, and is the edited text is added at Page 13, lines 259-270

**Query 5:** Reference for lines 252-253

**Answer:** The reference # 19 & 23 is included, as suggested.

**Query 6:** *Please re-read for grammar edits pp. 12, line 255 insert comma (,) after “On the contrary,…”*

**Answer:** The sentence is edited, as suggested.

I hereby, certify that all the authors have reviewed and agreed to the revised version and answers to reviewer’s queries. We hope that the revised version of the manuscript will be acceptable for publication to the Journal PLOS ONE.

Regards

Dr. Jamshaid Iqbal

Corresponding Author
